# Global trends in health research and development expenditures – the challenge of making reliable estimates for international comparison

**DOI:** 10.1186/1478-4505-13-7

**Published:** 2015-01-24

**Authors:** Alison J Young, Robert F Terry, John-Arne Røttingen, Roderik F Viergever

**Affiliations:** Former OECD Directorate for Science, Technology and Industry, Paris, France; TDR, the Special Programme for Research and Training in Tropical Diseases, Geneva, Switzerland; Division of Infectious Disease Control, Norwegian Institute of Public Health, Oslo, Norway; Department of Health Management and Health Economics, Institute for Health and Society, University of Oslo, Oslo, Norway; Department of Global Health and Population, Harvard T.H. Chan School of Public Health, Boston, MA USA; Radboud Institute for Health Sciences, Radboud University Medical Center, Nijmegen, The Netherlands; Department of Health Services Research and Policy, London School of Hygiene and Tropical Medicine, London, UK

**Keywords:** Health financing, Health research, Research and development, Research and development deflators, Research and development exchange rates, Research funding, Research methods

## Abstract

Better estimates of changes in the level and structure of national, regional, and global expenditures on health research and development (R&D) are needed as an important source of information for advancing countries’ health research policies. However, such estimates are difficult to compile and comparison between countries needs careful calibration. We outline the steps that need to be taken to make reliable estimates of trends in countries’ expenditures on health R&D, describe that an ideal approach would involve the use of international sets of deflators and exchange rates that are specific to health R&D activities, and explain which methods should be used given the current absence of such health R&D-specific deflators and exchange rates. Finally, we describe what should be the way forward in improving our ability to make reliable estimates of trends in countries’ health R&D expenditures.

## Difficulties in comparing countries’ health-related R&D expenditures

Estimates of changes in the level and structure of national, regional, and global expenditures on health research and development (R&D) are an important source of information for advancing countries’ health research policies. However, such estimates are difficult to compile and comparison between countries needs careful calibration [1].

At present, the availability of user-friendly guidelines on how to compile and compare such data is limited outside two technical annexes to the international standard practice for surveys of R&D known as the Frascati Manual (one on identifying health R&D spending and the other on R&D deflators and currency converters) [2, 3]. These annexes are not always identified by online searches and are essentially written by and for the experts who collect R&D statistics, rather than for analysts from within the health research system who want to use them.

The difficulties concerned can be illustrated by a comparison between two recent efforts to compile and compare national contributions to world-wide expenditures on health-related R&D. Chakma et al. [4] recently published an article comparing decreases in spending on health R&D in the United States between 2007 and 2012 to increases in countries in Asia-Oceania in a world-wide context. The estimates developed by Chakma et al. [4] appear to differ markedly from our own published findings of an extensive mapping of all countries’ expenditures on health R&D in 2009, which also examined the distribution between low-, middle-, and high-income countries [5]. Here, we set out how these differences occurred and identify the topics on which guidance is needed.

Exercises to estimate countries’ expenditures on health R&D involve two stages. First, national and international sources of relevant data need to be tracked down and the health-related categories of expenditure extracted. Second, these health-related expenditures need to be converted to comparable price levels over time using a common currency between countries. The data sources used in our own recent study varied in some cases from those used by Chakma et al. [4], but it is at the second stage – data conversion – that the main differences arose.

## Absence of relevant price indices and currency convertors

To convert countries’ health-related R&D expenditures to comparable price levels, ideally one needs exchange rates that are specific to the “basket of goods” used in health R&D activities. To be able to compare these expenditures over time also requires indices that describe how the price of that basket of goods deflates or inflates over time in each country. Unfortunately, there are currently no standard international sets of deflators or exchange rates based on the basket of goods used in R&D activities. As a result, there is no choice but to choose proxy series of price indices and currency convertors, and to opt for the “least unsatisfactory” approach. For countries with developed sets of R&D statistics, health accounts, and general economic statistics, there is a range of possible proxies from which to choose. However, for a world-wide comparison, one needs to select proxy series which are available for all countries, thus reducing the available options considerably. In this article, we describe what the “least unsatisfactory” option is for converting health-related R&D expenditures to one common currency and for comparing these expenditures over time.

## Converting health-related R&D expenditures to one common currency

When comparing health R&D expenditures between countries they must be put in a common currency. In the absence of special “health R&D exchange rates” based on the comparative costs of the inputs into the activity, the two conversion rates most readily available are current exchange rates and GDP purchasing power parities (PPPs). Current exchange rates mainly reflect the cost of traded goods and services rather than domestic activities such as R&D and may also be affected by currency speculation, political events, and government controls (Sidebar on “Comparing international R&D expenditures” in [6]). GDP PPPs, on the other hand, were developed to provide better convertors for comparisons of national wealth as measured by GDP [7]. A relatively recent detailed study on the topic showed that these GDP PPPs do not reflect the cost of R&D very accurately as compared with estimated R&D PPPs [8]. However, the study covers only the manufacturing industry in six developed countries and two years (1987 and 1997). For world-wide comparisons one must still make a “least unsatisfactory” choice between current exchange rates and GDP PPPs. The WHO health accounts database offers both options for total health expenditures. Chakma et al. [4] chose current exchange rates for their main tables; we followed the recommendation of the Frascati manual and used GDP PPPs (as Chakma et al. did in a pie-chart in the online annex to their paper [4]). Using current exchange rates instead of PPPs underestimates the contribution of countries such as China and India, because, for the reasons given above, exchange rates in these countries overstate the cost of domestic activities and thus probably of R&D.

## Comparing trends in health R&D expenditures over time

When comparing trends in health R&D expenditures over time in different countries it is necessary to adjust the current price data in order to exclude the contribution of inflation to apparent growth. To do so, one needs an index of the trend in the price of the inputs into health-related R&D in each country. The only long established example of such an index is the special R&D deflator used in the annual discussions of the budget of the US National Institutes of Health (NIH) [9]. It, like other estimates of trends in R&D costs in developed countries [10], shows that the price of the “basket of goods” used in R&D grows at a different rate, usually higher, than the average of prices included in the implicit GDP price index. In the absence of a specific health R&D index, the main options for other countries are to use the implicit GDP price index, which is currently recommended by the Frascati manual, or to use the consumer price index, which suffers from the same defects as the GDP deflator for R&D analysis purposes (i.e., that it is not specific to R&D). Chakma et al. [4] chose to deflate the health R&D expenditures of all countries using the US NIH price index. This is questionable as the general level of inflation varies between countries more than the difference between the R&D and the general price index in the United States. Thus, the use of the NIH R&D price index flatters countries with high inflation, such as India, and underestimates growth in health R&D funding in countries where general prices were stagnant, such as Taiwan.

## Combining prices and currency convertors

Finally, in this exercise we need to apply the price indices and currency converters we have selected to our original expenditure series in national currency. These must be combined in a way which avoids inappropriate interactions between the price index and the currency converter. The established method is to first deflate the national currency data and then to convert the deflated data for all years to a common currency using an appropriate exchange rate for the base year for the price index. In the study by Chakma et al. [4], the data were first converted year by year to US dollars and then deflated using 2012 as the base year. This method overestimates growth in funding in countries such as Japan, where R&D spending grew little in national currency between 2007 and 2012 while the value of the yen appreciated. Additionally, it underestimates the increase in countries such as South Korea where the national currency fell against the dollar over the same period.

## Effect of using different analytical methods and data sources to compare trends in health-related R&D between the USA and Oceania

Using data derived from the text table in the article by Chakma et al. [4], we have recalculated the health R&D expenditures for the USA and Asia-Oceania using the methods described above, which should be considered current best practice (Table 1). Each stage of this re-calculation has different impacts on each country. Ultimately, the gross increase in health R&D expenditure in fixed price PPP dollars in the Asia-Oceania zone is only slightly lower than in the original article by Chakma et al. [4]. However, the balance between the countries clearly changes. China now has the largest growth, followed by South Korea. In India growth is higher, but in Japan and Australia less. These results support the authors’ thesis, though the decline in the United States is not so marked.

**Table 1:** C**hanges in estimated health R&D spending between 2007 and 2012 for countries in Asia-Oceania, the USA and Canada, using two methods and two data sources (in billion US dollars)**

Country	Data from Chakma et al. [4]		Data from Røttingen et al. [5]	
	Current exchange rates and NIH 2012 prices	2012 PPP exchange rates and 2012 GDP prices	Current exchange rates and NIH 2012 prices	2012 PPP exchange rates and 2012 GDP prices
USA	−12.0	−4.0	– *	
Canada	−0.7	−0.7	−1.0	−1.0
Asia-Oceania	20.9	19.2	17.0	15.0
Japan	9.0	2.8	7.3	2.4
China	6.4	8.7	4.3	5.6
South Korea	2.5	4.3	2.2	3.9
Australia	1.7	0.4	2.2	0.8
India	0.6	1.6	0.5	1.4
Other Asia-Pacific**	0.6	0.7	0.5	0.9

Column 1 is the original data from Chakma et al. [4]. In column 2, we have applied analytical approaches to their data that are currently considered best practice. In columns 3 and 4 we applied both their method and the best practice approaches to time series compiled on the same basis as the 2009 data in our own study, which draw on different data sources in some cases. [5].

*We were unable to estimate the growth in health related R&D expenditure in the United States on the same basis as we used in our article as the underlying figures for industry were only collected for 2008 and 2009 [11].

**Column 2 is recalculated from the data for Singapore and Taiwan in the pie-chart in the appendix of the article by Chakma et al. [4] combined with the growth rates in the text chart as the two countries are grouped in the text table used for the other countries.

Besides different approaches to analysis, the use of different data sources can also lead to diverging estimates of countries’ health R&D expenditures. To see whether the use of an alternative dataset changes the results of the analysis, we have also calculated the trends in health R&D expenditures in the countries in Asia-Oceania using our data [5] instead of Chakma et al.’s [4], and applying both their method and the best practice methods of deflation and currency conversion (Table 1). This analysis confirms the growth in health R&D expenditures in Asia-Oceania, though in most countries at a slightly lower level. This difference likely stems from our data being derived from standard surveys of the performers of R&D (supplementary appendix to [5]), whose results tend to change less rapidly than data taken from the reports of funding bodies used in a number of cases by Chakma et al. (supplementary table one in [4]). The reasons for differences between data derived from funder and performer reported sources are described in the Frascati Manual [12].

## Conclusions

In conclusion, estimates of national and global expenditures on health R&D can vary significantly depending on the methods that are used in the analysis. Making reliable estimates is currently hampered by the absence of standard health R&D price indices and exchange rates, though general work to redress this absence is currently in progress in the context of treating R&D as an intangible investment in the latest edition of the System of National Accounts [13]. Hopefully, following the recent decision by WHO Member States to establish a Global Health R&D Observatory, progress will be made in the future on developing and agreeing on such standards [14, 15]. This will be important if we are to make progress in generating data and indicators that are truly comparable and capable of influencing countries’ health research policies. In the meantime, in the absence of R&D-specific price indices and exchange rates, the development of user-friendly interim guidelines on selecting and applying the available price indices and convertors would be a useful first step and a potential output from the newly established Observatory [14].

## Abbreviations

NIH US: National Institutes of Health
PPPs: Purchasing power parities
R&D: Research and development.
